# AI-Powered Discovery of Rosmarinic Acid as a Novel Ferroptosis Inhibitor for Ulcerative Colitis via Targeting the ALOX15–VDAC1 Axis

**DOI:** 10.34133/research.1352

**Published:** 2026-07-08

**Authors:** Guixuan Fang, Jingli Ao, Xiao Li, Weiwei Wang, Yamina Alioui, Yang Huang, Jiali Peng, Bufu Tang, Lianxiang Luo

**Affiliations:** ^1^ School of Ocean and Tropical Medicine, Guangdong Medical University, Zhanjiang, Guangdong 524023, China.; ^2^ Department of Interventional Radiology, Zhongshan Hospital, Fudan University, Shanghai 200032, China.

## Abstract

Ulcerative colitis (UC) is a chronic inflammatory bowel disease with limited mechanism-based therapies, and ferroptosis—an iron-dependent lipid peroxidation-driven cell death—has emerged as a critical pathogenic event. Using a machine learning model that combines molecular fingerprints and transfer learning representations to screen for ALOX15 inhibitors, we identified rosmarinic acid (RA) from natural product libraries. RA exhibited high-affinity binding to ALOX15 and maintained stable complex conformation in molecular dynamics simulations. In dextran sulfate sodium-induced colitis mice and lipopolysaccharide-treated intestinal epithelial cells (IEC-6 and mouse colonic epithelial cell), RA alleviated disease activity index, colon shortening, histological damage, and barrier protein loss (ZO-1, Occludin, and Claudin-1). Mechanistically, RA suppressed ferroptosis by restoring GPX4, SLC7A11, and FTH1; reducing ACSL4, COX-2, and lipid reactive oxygen species (ROS); and lowering intracellular Fe^2+^ and malondialdehyde. Crucially, RA disrupted the ALOX15–VDAC1 protein complex, preserved mitochondrial membrane potential, and reduced mitochondrial ROS, effects that were abrogated by ALOX15 knockdown or VDAC1 knockdown. Serum metabolomics further revealed that RA remodels lipid metabolism pathways (arachidonic acid and linoleic acid metabolism) linked to ferroptosis. Collectively, this artificial intelligence-driven discovery positions RA as a novel ferroptosis inhibitor that targets the ALOX15–VDAC1 axis, offering a promising therapeutic candidate for UC.

## Introduction

Ulcerative colitis (UC) is a chronic relapsing inflammatory bowel disease characterized by mucosal inflammation of the colorectal epithelium. Its global burden continues to rise, particularly in newly industrialized countries, where incidence rates have increased markedly in recent decades [1]. Clinically, UC presents with heterogeneous manifestations; rectal bleeding or hematochezia is a hallmark feature, reported in nearly 90% of cases, and is frequently accompanied by abdominal pain, fatigue, and systemic symptoms that markedly impair patients’ health-related quality of life. At the mechanistic level, dysfunction of the intestinal epithelial barrier is widely recognized as an early and pivotal event in UC pathogenesis. Despite considerable advances, the molecular underpinnings of UC disease progression remain incompletely understood, thereby limiting the rational development of mechanism-based therapeutics [2,3].

In parallel, machine learning-assisted virtual screening has emerged as a useful computational strategy for prioritizing bioactive compounds from large chemical libraries. Such approaches can improve the efficiency of candidate prioritization and support early-stage lead identification. Notably, machine learning-based screening approaches have shown utility in exploring the structural complexity and pharmacological diversity of natural products [4,5]. These advances collectively support the application of machine learning-assisted virtual screening as a candidate-prioritization strategy for identifying bioactive natural products of potential relevance to UC.

Ferroptosis is a regulated, iron-dependent form of cell death characterized by the accumulation of lipid peroxides and disrupted metabolism of polyunsaturated fatty acids [6–8]. Accumulating evidence indicates that ferroptosis is implicated in the pathogenesis of numerous diseases [9], including malignant tumors, metabolic disorders such as diabetes, and autoimmune conditions such as psoriasis [10–17]. Importantly, experimental inhibition of ferroptosis has been shown to attenuate disease progression in animal models. Of particular relevance to UC, emerging studies indicated that ferroptotic death of intestinal epithelial cells contributes critically to barrier disruption and mucosal inflammation, thereby accelerating disease onset and exacerbation.

Rosmarinic acid (RA) is a naturally occurring polyphenol [18] with a favorable safety profile, which has demonstrated therapeutic potential in a variety of cancers [19–27] and exhibits gastroprotective effects in animal models [20–28]. Recent studies also showed that RA can reverse chemotherapy resistance and mitigate treatment-induced toxicity [29–31]. Despite these promising findings, its therapeutic potential and underlying mechanisms in UC remain to be established.

In this study, we employed a comprehensive multidisciplinary strategy. First, we applied a machine learning model to perform virtual screening of a natural product library, which rapidly identified RA as a promising candidate with high-affinity binding potential to ALOX15. Thereafter, through molecular docking, molecular dynamics (MD) simulations, and a series of in vitro and in vivo experiments, we found that RA alleviates UC through inhibition of ALOX15 and suppression of ferroptosis in intestinal epithelial cells. This work not only identifies a novel drug candidate for UC therapy but also underscores the practical value of an artificial intelligence-driven, experimentally validated research paradigm in translational medicine.

## Results

### ALOX15 is up-regulated in UC and exhibits diagnostic potential, correlating with inflammatory and metabolic reprogramming

**Fig. 1.**
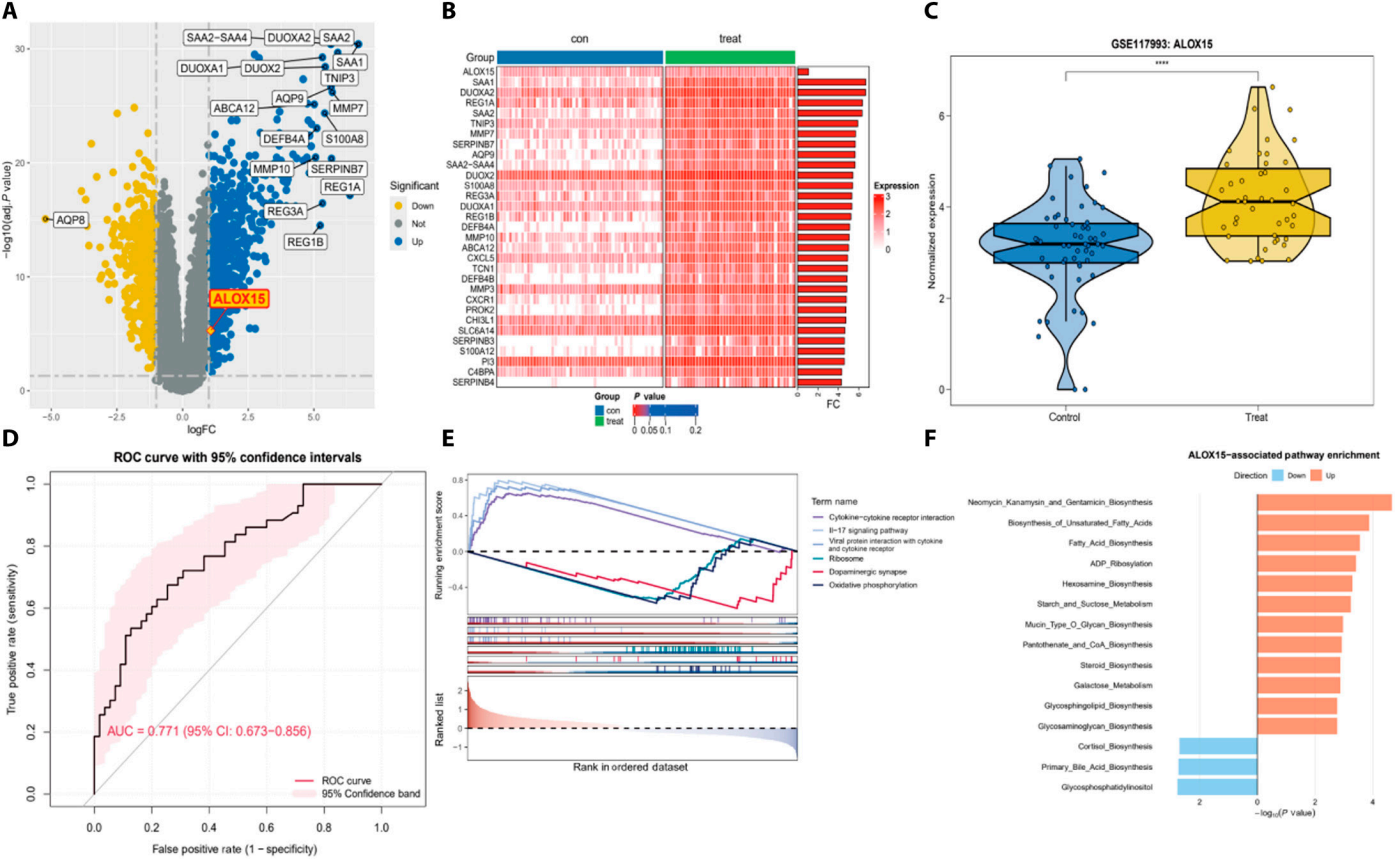
Differential identification of ALOX15 and exploration of biological functions. (A) Volcano plot indicating DEGs based on statistical significance and fold change, with |log_2_FC| > 1 highlighted and ALOX15 marked specifically. (B) Heatmap showing the expression profile of ALOX15 alongside the top 30 DEGs. (C) Violin-boxplot illustrating the expression differences of ALOX15 between healthy and UC groups. (D) ROC curve depicting the diagnostic ability of ALOX15 for sample classification. (E) GSEA displaying the top 3 and bottom 3 biological pathways based on KEGG-derived NES values. (F) GSVA bar graph comparing enrichment score differences across 114 metabolic pathways between high- and low-expression groups.

Analysis of differentially expressed genes (DEGs) between UC and control samples in the GSE117993 dataset identified 1,262 DEGs meeting the criteria of |log_2_FC| > 1 and *P* < 0.05 (Fig. 1A and B) including the core gene ALOX15. Furthermore, relative to controls, ALOX15 expression was markedly elevated in the UC group (Fig. 1C). Receiver operating characteristic (ROC) curve analysis demonstrated that ALOX15 expression effectively discriminates UC samples from healthy controls, with an area under the curve (AUC) of 0.771 (Fig. 1D), indicating that it has good diagnostic potential. Gene set enrichment analysis (GSEA) revealed that high ALOX15 expression is associated with substantial up-regulation of inflammation-related pathways (especially cytokine signaling), while basal metabolism and protein synthesis-related pathways are markedly down-regulated (Fig. 1E). In the ALOX15 high-expression group, the pathway of positive enrichment includes cytokine–cytokine receptor interaction, which exhibited the highest enrichment, indicating activation of a cytokine- and receptor-mediated inflammatory signaling network. The interleukin-17 (IL-17) signaling pathway is also substantial ly enriched, suggesting that Th17 cell activity and IL-17 secretion in UC patients are associated with ALOX15 expression levels. The crosstalk between viral proteins and cytokine receptor pathways also implies that elevated ALOX15 expression may be linked to altered viral–cytokine interactions and disrupted intestinal barrier and mucosal immune function in UC.

Gene set variation analysis (GSVA) further revealed that high ALOX15 expression is associated with up-regulation of lipid biosynthesis and coenzyme A, while the down-regulated metabolic pathway involves bile acid, carbohydrate metabolism, and mitochondrial energy metabolism (Fig. 1F). Up-regulated pathways include sphingolipid biosynthesis, fatty acid biosynthesis, and unsaturated fatty acid biosynthesis. Notably, in both GSEA and GSVA, the oxidative phosphorylation pathway was negatively correlated with high ALOX15 expression, suggesting an association with mitochondrial dysfunction in the inflammatory microenvironment. This metabolic reprogramming pattern may shed new light on the mechanistic involvement of ALOX15 in the pathogenesis and progression of UC.

### Machine learning identifies RA as a potential ALOX15 inhibitor

**Fig. 2.**
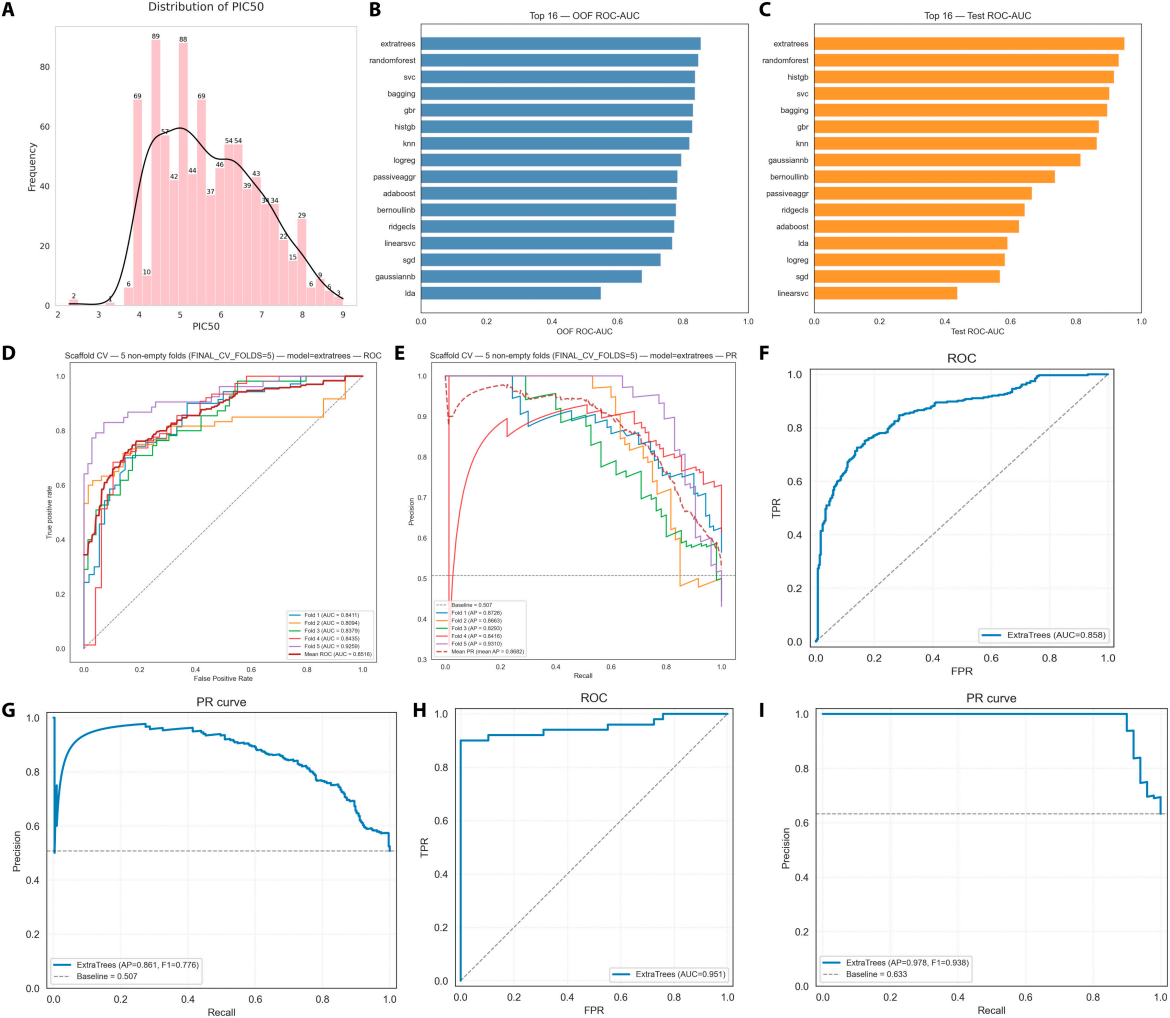
Machine learning results for the ALOX15 inhibitor dataset. (A) Distribution of pIC_50_ values in the ALOX15 inhibitor dataset. (B) Ranking of classification models by OOF ROC-AUC values. (C) Ranking of classification models by Test ROC-AUC values. (D) ROC curves for each fold of ExtraTrees 5-fold cross-validation training. (E) PR curves for each fold of ExtraTrees 5-fold cross-validation training. (F) ROC curve of ExtraTrees with single-model nested optimization on OOF. (G) PR curve of ExtraTrees with single-model nested optimization on OOF. (H) ROC curve of ExtraTrees with single-model nested optimization on the independent test set. (I) PR curve of ExtraTrees with single-model nested optimization on the independent test set.

Prior to modeling, standardized cleaning of the ALOX15 inhibitor data sourced from ChEMBL was performed. Simplified Molecular-Input Line-Entry System (SMILES) validity was first checked using RDKit, and invalid structures were removed. Subsequently, duplicate molecular entries were aggregated by mean activity values, reducing the dataset from 904 original records to 698 molecules suitable for modeling. Half maximal inhibitory concentration (IC_50_) values were converted to pIC_50_ [−log10(IC_50_)], and active compounds were defined as those with pIC_50_ ≥ 5.2 (corresponding to IC_50_ ≤ 6.3 μM) to construct a binary classification task. The resulting dataset showed a near-balanced class distribution in the scaffold-based train-development subset (positive prevalence ≈ 50.7%), whereas the independent scaffold-disjoint test set contained 63.3% active compounds. Detailed statistics of scaffold-level dataset partitioning are provided in Table S3. The activity distribution of the 698 ChEMBL compounds is shown in Fig. 2A.

The molecular features comprised a combination of molecular fingerprints and transfer learning representations (Morgan + Molecular ACCess System [MACCS] + pretrained representation). Specifically, approximately 3 million drug-like small molecules were randomly collected from the ZINC database for unsupervised pretraining without using any ALOX15 activity annotations. Because the pretraining stage was conducted exclusively on unlabeled external molecules, no activity information from the downstream ALOX15 dataset was involved during representation learning.

For all molecules, Morgan fingerprints (radius 2, 2,048 bits) and MACCS fingerprints (167 bits) were computed, followed by median imputation, low-variance filtering (threshold 0.16), and standardization. Finally, incremental principal component analysis (IPCA) was applied to compress the features into a 128-dimensional latent space. For the downstream ALOX15 classification task, each molecule was processed with the same fingerprint extraction and preprocessing pipeline, and the resulting features were concatenated with the 128-dimensional pretrained representation to form the final model input. This hybrid feature strategy enabled the model to capture both target-specific local structural signals and global chemical space priors derived from large-scale pretraining. This strategy substantially improves generalization robustness when using limited labeled samples.

Among the benchmarked models, ExtraTrees demonstrated superior performance in terms of generalization capability and stability (Fig. 2B and C). After single-model nested optimization, the final ExtraTrees model achieved ROC-AUC = 0.8584 (95% confidence interval [CI]: 0.829 to 0.889) (Fig. 2D) and precision-recall (PR)-AUC = 0.861 (95% CI: 0.826 to 0.901) (Fig. 2E) on scaffold 5-fold out-of-fold (OOF) validation. Additional OOF metrics included F1 = 0.776, Matthews correlation coefficient (MCC) = 0.574, balanced accuracy = 0.786, precision = 0.822, and recall = 0.736 (threshold = 0.48, selected exclusively from OOF predictions). The 5-fold cross-validation ROC curves (Fig. 2F) and PR curves (Fig. 2G) showed tightly clustered profiles with minimal AUC variation across folds, indicating good predictive consistency and stability across different structural subsets. Class distributions across the outer validation folds remained relatively balanced (Table S5). On the independent scaffold test set, the model reached ROC-AUC = 0.9510 (95% CI: 0.898 to 0.994) (Fig. 2H) and PR-AUC = 0.9783 (95% CI: 0.952 to 0.995) (Fig. 2I). Corresponding test-set metrics included F1 = 0.938, MCC = 0.8459, balanced accuracy = 0.9328, precision = 0.9783, and recall = 0.9000, using the unchanged OOF-derived threshold (0.48). These results support the model’s discriminative capability under scaffold-disjoint evaluation settings. However, because the independent holdout subset contained a relatively limited number of scaffold series, the broader assessment of scaffold-level generalization primarily relied on scaffold-grouped OOF validation across the train-development subset. Nevertheless, because the independent scaffold test set was relatively small (*n* = 79; 3 Murcko scaffolds), the observed test performance should be interpreted cautiously. In addition, Y-randomization analysis (499 label permutations) yielded a null ROC-AUC distribution centered near random expectation (mean ROC-AUC = 0.494) (Table S8), substantially lower than the observed model performance, supporting that the predictive signal was unlikely to arise from chance correlations.

Following priority screening of the candidate antioxidant small molecule library using the final model, 41 small molecules predicted to be active were obtained (Table S1). Molecular docking was performed on these small molecules, and the docking results (Table S2) showed the docking scores of the candidate molecules, among which RA achieved the highest docking score of −7.22 kcal/mol.

### The impact of RA on the serum metabolic profile of C57 mice

To gain deeper insight into the metabolic alterations induced by RA treatment in C57 mice, differential metabolite analysis was performed.

**Fig. 3.**
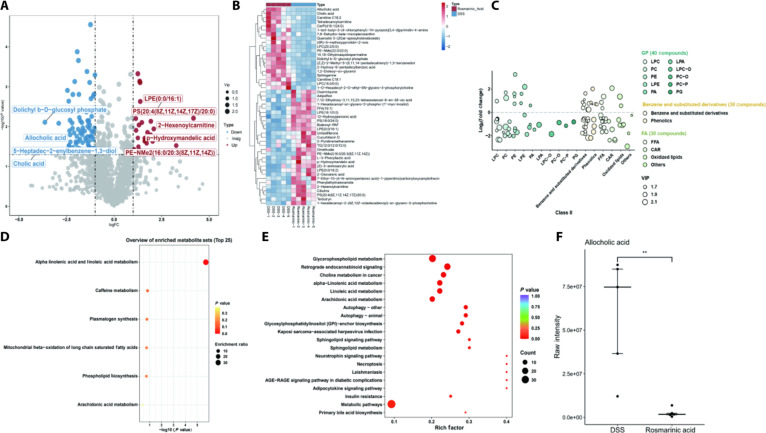
The effect of rosmarinic acid on the serum metabolic profile of C57 mice. (A) Volcano plot of differential metabolites (rosmarinic acid vs. DSS). Red circles represent markedly up-regulated metabolites, and blue circles represent markedly down-regulated metabolites. (B) Heatmap displaying the top 48 differential metabolites in both groups (VIP > 1 and *P* < 0.05). (C) The bubble plot of metabolite classification shows the categories of the differential metabolites and the distribution of their VIP values. (D) SMPDB enrichment analysis of markedly differential metabolites through MetaboAnalyst. (E) KEGG pathway enrichment bubble chart. (F) Metabolites that have markedly changed after administration.

The volcano plot revealed 29 markedly up-regulated metabolites and 86 markedly down-regulated metabolites (Fig. 3A). Between the RA and dextran sulfate sodium (DSS) groups, 48 major metabolites exhibiting significant differences were screened out (variable importance in the projection [VIP] > 1, *P* < 0.05) (Fig. 3B). Classification of the markedly altered metabolites showed that within the major categories of glycerophospholipids and fatty acids, the number of down-regulated metabolites exceeded that of up-regulated ones (Fig. 3C). Small Molecule Pathway Database (SMPDB) enrichment analysis indicated that RA influenced lipid metabolism, including alpha linolenic acid and linoleic acid metabolism, mitochondrial beta-oxidation of long-chain saturated fatty acids, and arachidonic acid metabolism (Fig. 3D). Additionally, RA was associated with caffeine metabolism, suggesting a potential link to drug metabolism. Kyoto Encyclopedia of Genes and Genomes (KEGG) enrichment analysis corroborated these findings and further revealed associations between RA and pathways such as Necroptosis and Autophagy (Fig. 3E). Furthermore, substantial changes in several metabolites associated with reactive oxygen species (ROS) suppression were also observed following RA administration (Fig. 3F). Therefore, we propose that RA is closely associated with lipid peroxidation and ferroptosis in UC.

### Computational and experimental validation of the direct binding interaction between RA and ALOX15

**Fig. 4.**
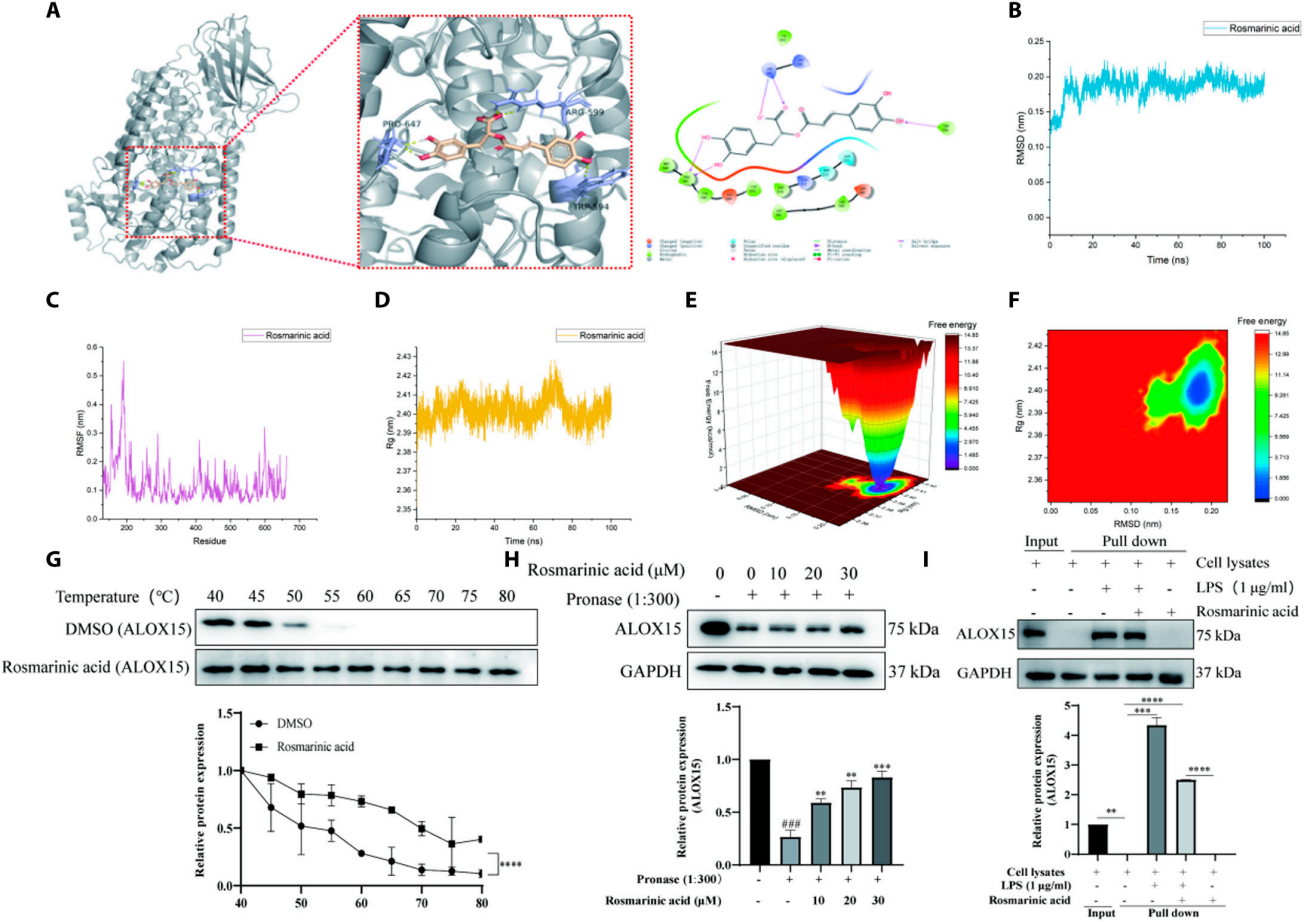
Validation of ALOX15 as a direct therapeutic target of rosmarinic acid in ulcerative colitis. (A) The binding mode of ALOX15 and the compound rosmarinic acid. (B) Analysis of the root mean square deviation (RMSD) of the ALOX15–rosmarinic acid complex. (C) Root mean square fluctuation (RMSF) of ALOX15–rosmarinic acid complex. (D) Radius of gyration (Rg) of the ALOX15–rosmarinic acid complex. (E) 3D diagram of the free energy landscape (FEL) of the ALOX15–RA complex. (F) FEL 2D diagram of the ALOX15–rosmarinic acid complex. (G) Cellular thermal shift assay (CETSA) showing that ALOX15 protein stability was enhanced by rosmarinic acid in MCECs. MCEC lysates were treated with DMSO or 30 μM rosmarinic acid and heated at the indicated temperatures (40 to 80 °C). (H) Drug affinity responsive target stability (DARTS) assay demonstrating that rosmarinic acid protected ALOX15 from pronase degradation in a concentration-dependent manner (0, 10, 20, and 30 μM). (I) Competitive pull-down assay confirming the direct interaction between rosmarinic acid and ALOX15 protein in MCEC lysates. Input: total cell lysates; Pull-down: eluted proteins from the pull-down assay. Compared with the control group, #*P* < 0.05, ##*P* < 0.01, ###*P* < 0.001, ####*P* < 0.0001. Compared with the Pronase group, *P < 0.05, **P < 0.01, ***P < 0.001, ****P < 0.0001.

To elucidate the molecular basis of ligand recognition, molecular docking simulations were conducted to characterize the binding interface between ligands and target proteins. This method provides insight for drug design and is a key tool for early screening of candidate drugs. Molecular docking predicted the high-affinity binding mode of RA in the active site of ALOX15, with a GlideScore of −7.219 kcal/mol. As shown in Fig. 4A, RA forms hydrogen bonds with TRP594 and PRO647, and additional interactions including hydrogen bonding and a critical salt bridge with ARG599 were identified, collectively indicating a stable binding interaction between RA and ALOX15. As shown in Fig. 4B, the root-mean-square deviation (RMSD) analysis of the 100-ns MD trajectory demonstrates that the average RMSD of the ALOX15–RA complex is 0.1846 nm, indicating that the overall conformational fluctuation of the protein after ligand binding is relatively small, which shows that the ALOX15–RA complex maintains high structural integrity throughout the simulation process. The root-mean-square fluctuation (RMSF) diagram of protein residues (Fig. 4C) further reveals the substantial limitation of atomic movement in the ALOX15 binding pocket, with an average RMSF of 0.1240 nm. Notably, the RMSF value at the key active-site residue PRO647 was markedly below the mean, indicating that the binding site has a high degree of structural rigidity. The analysis of the radius of gyration (Rg) (Fig. 4D) shows that the average Rg value of the complex is 2.4019 nm, and the fluctuation is less than 0.08 nm throughout the simulation, confirming that the protein maintains a tight folded configuration without obvious structural loosening. Free energy landscape (FEL) analysis revealed that through the 3-dimensional drawing of RMSD–Rg free energy value (Fig. 4E and F), the conformational space of the ALOX15–RA complex presents a unique deep and wide energy well distribution, which may be an important dynamic basis for supporting a favorable free energy of binding. To assess the interaction and stability between RA and ALOX15, we employed dimethyl sulfoxide (DMSO) or protein extracts from RA-treated mouse colonic epithelial cells (MCECs) for cellular thermal shift assay (CETSA)–Western blot analysis. The results showed that ALOX15 exhibits high thermal stability in the RA treatment group. Drug affinity responsive target stability (DARTS) experiments showed that RA inhibits the protein degradation of ALOX15 through affinity protease. In addition, a competitive pull-down experiment confirmed the interaction between RA and ALOX15 protein (Fig. 4G to I). Collectively, these computational and in vitro binding data support ALOX15 as a key functional protein regulated by RA.

### RA ameliorates DSS-induced experimental colitis and intestinal epithelial barrier dysfunction by targeting ALOX15

**Fig. 5.**
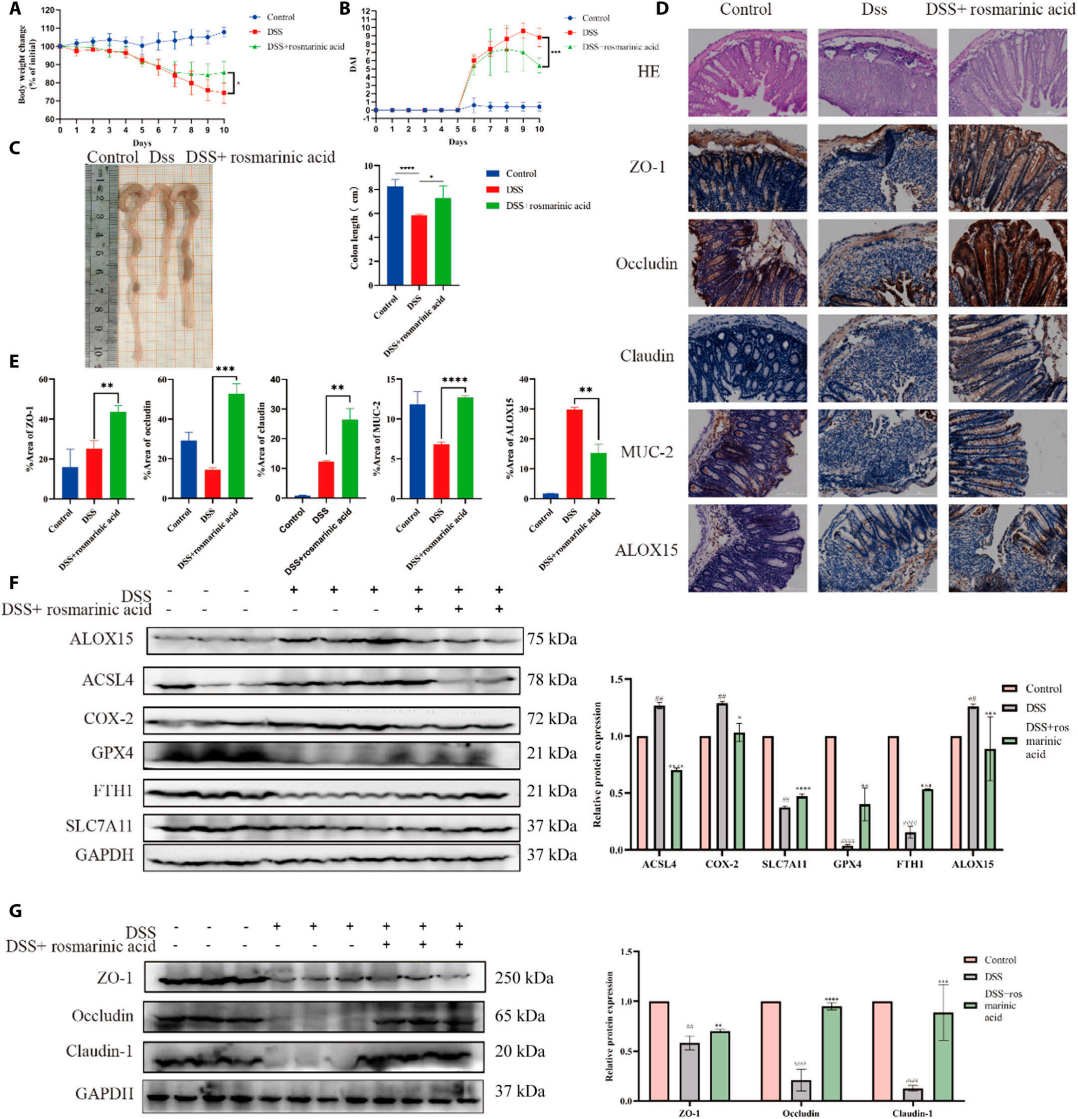
Attenuation of DSS-induced colitis by rosmarinic acid through suppression of ALOX15-dependent ferroptosis and preservation of intestinal barrier function. (A) The daily weight change of each group of mice. (B) Disease activity index (DAI) score of each group of colitis mice. (C) Representative images of colons from each group. (D) Representative H&E and immunohistochemical staining images of colon tissues showing the expression of intestinal barrier proteins in each group. Image captured at ×20 magnification; scale bar: 100 μm. (E) Quantitative analysis of the expression levels of intestinal barrier proteins (ZO-1, Occludin, Claudin-1, and MUC-2) and ALOX15 using ImageJ software based on previously shown IHC staining images. (F) Relative protein expression levels of ferroptosis-related markers (ACSL4, COX-2, ALOX15, GPX4, FTH1, and SLC7A11) in mouse colon tissue assessed by Western blotting and quantified using ImageJ software. (G) Relative protein expression levels and quantitative analysis of tight junction proteins (ZO-1, Occludin, and Claudin-1). Compared with the DSS group: *P < 0.05, **P < 0.01, ***P < 0.001, ****P < 0.0001.

To investigate the therapeutic potential of RA in UC, a mouse model was established using 3% DSS. Compared with the DSS group, both pretreatment and combined treatment with RA (100 mg/kg) substantially improved disease severity by attenuating weight loss, reducing colon shortening, and lowering disease activity index (DAI) score (Fig. 5A to C). Histological examination (hematoxylin and eosin [H&E] staining) showed that RA substantially improved colonic pathology and reduced inflammatory cell infiltration, mucosal damage, and goblet cell loss (Fig. 5D). Immunohistochemistry further demonstrated that RA treatment effectively down-regulated the expression of ALOX15 and up-regulated key intestinal barrier proteins, including ZO-1, Occludin, Claudin-1, and MUC-2 (Fig. 5D and E).

To further characterize the underlying mechanism, protein expression related to ferroptosis and epithelial barrier function was analyzed. Western blot results showed that DSS challenge promoted ferroptotic cell death, as indicated by increased ACSL4, ALOX15, and COX-2 alongside decreased GPX4, FTH1, and SLC7A11. RA administration effectively counteracted these protein expression changes (Fig. 5F). Additionally, tight junction proteins including ZO-1, Occludin, and Claudin-1, which were markedly reduced by DSS, were restored upon RA treatment, reflecting restoration of epithelial barrier function (Fig. 5G).

### RA inhibited ferroptosis in LPS-treated IEC-6 and MCEC

**Fig. 6.**
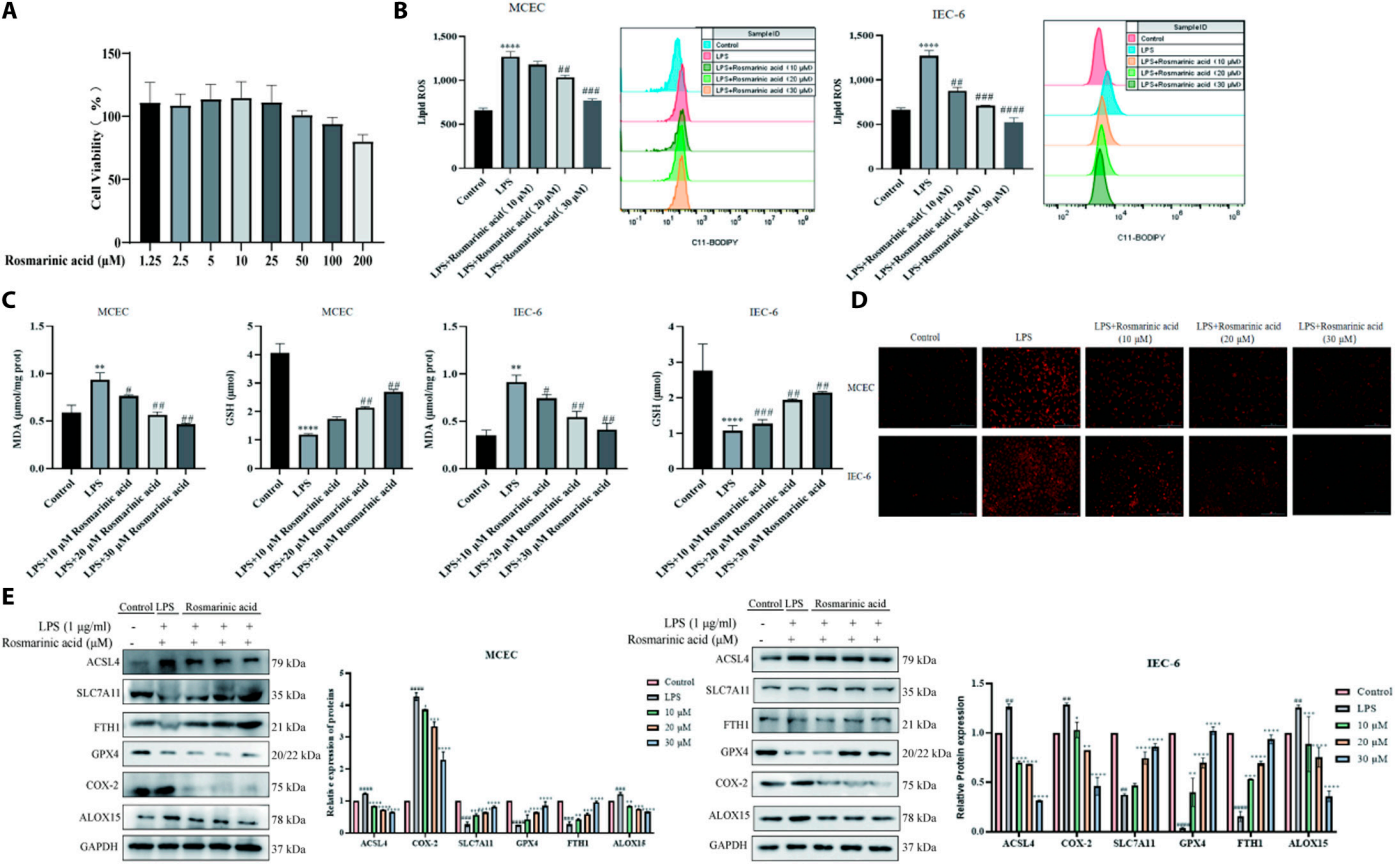
Rosmarinic acid suppresses LPS-triggered ferroptosis in intestinal epithelial cells under in vitro conditions. (A) Cell viability of MCECs treated with different concentrations of rosmarinic acid (1.25 to 200 μM) for 24 h, determined by CCK-8 assay. (B) Flow cytometry detection of lipid peroxidation levels: LPS markedly promotes lipid ROS production, while rosmarinic acid reduces it. (C) Rosmarinic acid markedly reduces malondialdehyde (MDA) levels and increases glutathione (GSH) levels in a concentration-dependent manner in vitro. (D) Intracellular Fe^2+^ content in IEC-6 and MCECs detected using a FerroOrange fluorescent probe and Cytation 5 (scale bar: 100 μm). (E) Western blot analysis showing that rosmarinic acid up-regulates GPX4, SLC7A11, and FTH1 expression in IEC-6 and MCECs in a concentration-dependent manner, while down-regulating ALOX15, ACSL4, and COX-2. Compared with the control group: #*P* < 0.05, ##*P* < 0.01, ###*P* < 0.001, ####*P* < 0.0001. Compared with the LPS group: *P < 0.05, **P < 0.01, ***P < 0.001, ****P < 0.0001.

The capacity of RA to counteract ferroptosis was assessed through a battery of cell-based experiments. A Cell Counting Kit-8 (CCK-8) viability assay first established that RA concentrations up to 30 μM had no measurable effect on MCEC survival over 24 h (Fig. 6A), ruling out confounding cytotoxic effects. Protein expression profiling in both MCEC and IEC-6 cells revealed that RA dose-dependently reversed the lipopolysaccharide (LPS)-driven suppression of FTH1, SLC7A11, and GPX4, while simultaneously attenuating the LPS-induced rise in ACSL4, ALOX15, and COX-2 (Fig. 6D). On a biochemical level, LPS exposure substantially increased intracellular Fe^2+^, lipid ROS, and malondialdehyde (MDA), all of which are hallmark indicators of ferroptotic stress, while glutathione (GSH) levels dropped accordingly. Coadministration of RA brought all these parameters back toward control values (Fig. 6B to E). Taken together, the data point to RA as an effective suppressor of LPS-triggered ferroptosis across both cell lines tested.

### RA inhibits LPS-induced ferroptosis in an ALOX15-dependent manner

**Fig. 7.**
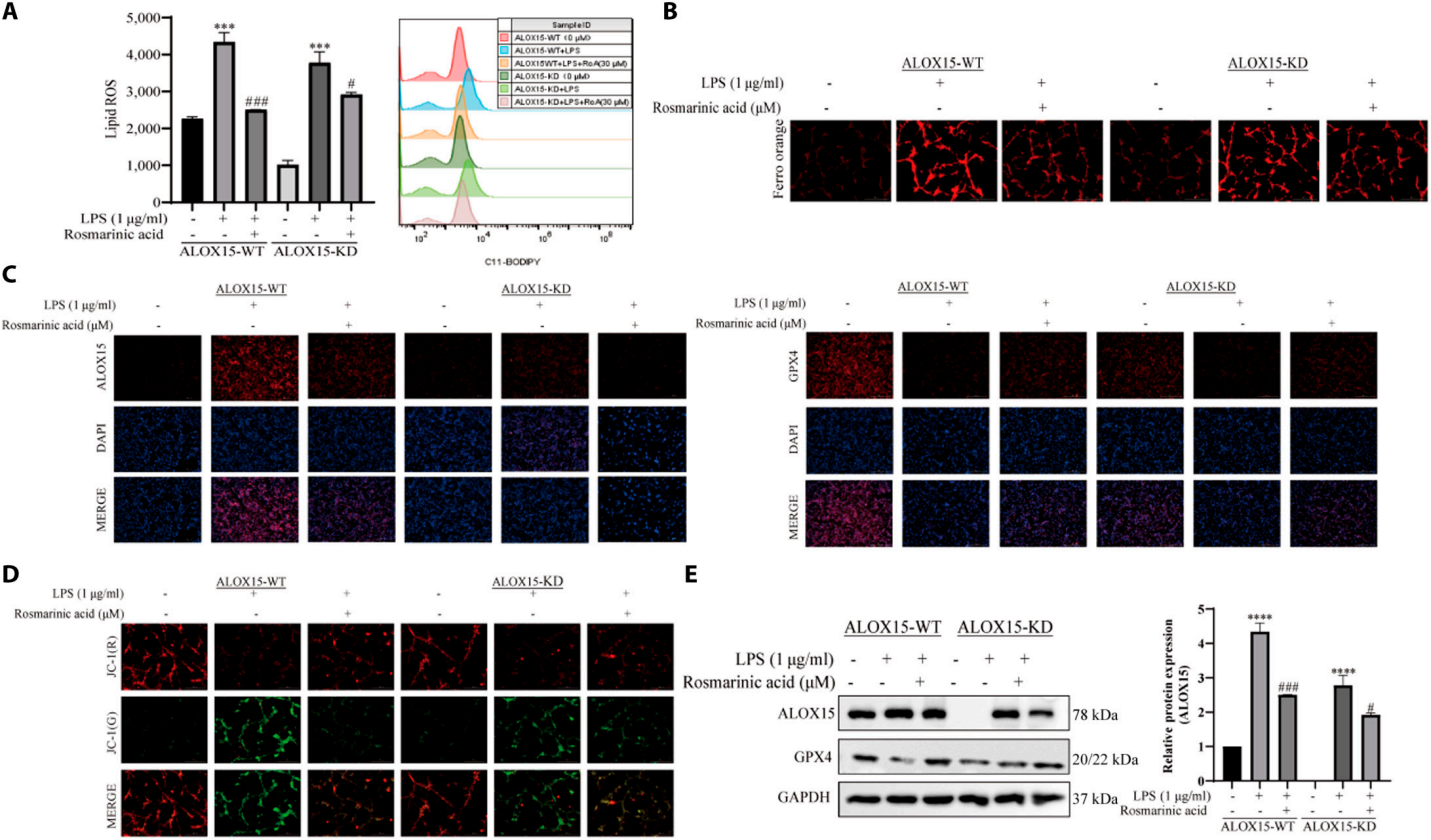
Rosmarinic acid attenuates inflammation through targeted suppression of ALOX15. (A) Rosmarinic acid does not reduce intracellular ROS production in ALOX15-KD cells. (B) Intracellular Fe^2+^ levels in ALOX15-KD cells. (C) Immunofluorescence images of ALOX15 and GPX4 in ALOX15-KD cells. (D) Mitochondrial membrane potential assessed by JC-1 staining (scale bar: 100 μm). (E) Western blot analysis of ALOX15 and GPX4 protein expression. Compared with the control group: #*P* < 0.05, ##*P* < 0.01, ###*P* < 0.001, ####*P* < 0.0001. Compared with the LPS group: *P < 0.05, **P < 0.01, ***P < 0.001, ****P < 0.0001.

To confirm that the protective effect of RA is mediated through ALOX15, experiments were conducted in ALOX15 knockdown (KD) cells. ALOX15 depletion abolished the ability of RA to rescue LPS-induced GPX4 down-regulation (Fig. 7A to E). Furthermore, in ALOX15-KD cells, the drug’s ability to inhibit intracellular Fe^2+^ accumulation was substantially diminished (Fig. 7B). Taken together, these data confirm that RA’s anti-ferroptotic protection is largely abrogated after ALOX15 depletion, establishing ALOX15 as a major effector of RA-mediated cytoprotection

### ALOX15 mediates the inhibitory effect of RA on LPS-induced ferroptosis in MCECs

**Fig. 8.**
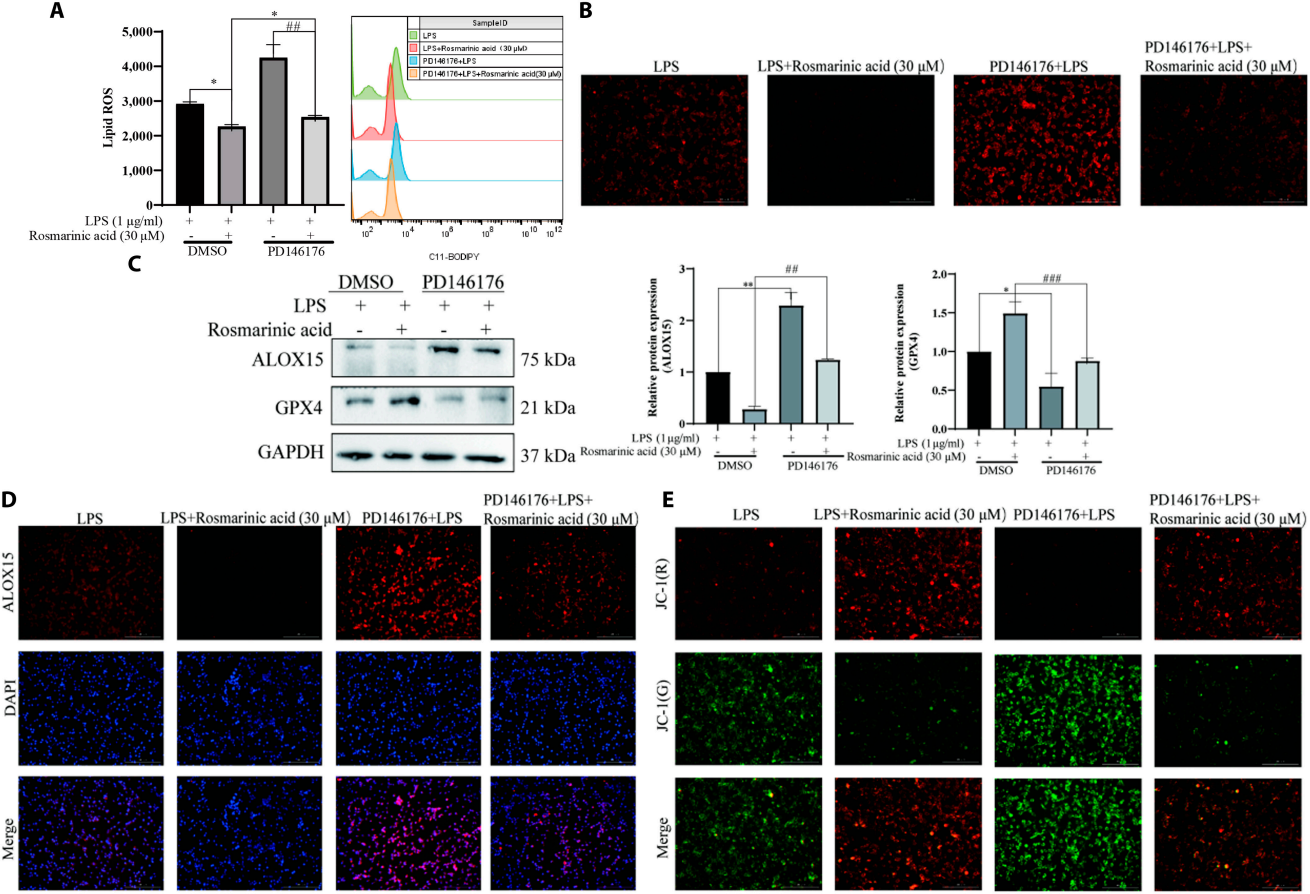
Role of the ALOX15 pathway in the protective effect of rosmarinic acid against colitis-associated inflammation. Cells were pretreated with rosmarinic acid (30 μM) for 2 h, followed by lipopolysaccharide (LPS; 1 μg/ml) treatment for 24 h. Prior to LPS treatment, cells were treated with DMSO or PD146176 for 1 h. (A) Assessment of lipid peroxidation by flow cytometry. (B) Detection of intracellular Fe^2+^ in MCECs using a FerroOrange fluorescent probe (scale bar: 100 μm; imaged with a Cytation 5 system). (C) Western blot analysis of ALOX15 and GPX4 protein levels following rosmarinic acid (30 μM) and LPS treatment (with or without PD146176), with quantification using ImageJ software. (D) Immunofluorescence imaging of ALOX15 expression and distribution in cells (scale bar: 100 μm). (E) Mitochondrial membrane potential assessed by JC-1 staining (scale bar: 100 μm). Data are expressed as mean ± SD; *P < 0.05, **P < 0.01, ***P < 0.001 compared with LPS-treated cells; #*P* < 0.05, ##*P* < 0.01, ###*P* < 0.001 compared with LPS + rosmarinic acid-treated cells.

To determine whether ferroptosis occurs in LPS-induced MCECs, we examined 3 key hallmarks: lipid peroxidation, Fe^2+^ accumulation, and antioxidant system dysfunction. Flow cytometry analysis revealed that lipid peroxidation levels were markedly elevated in cells treated with the ALOX15 inhibitor combined with LPS (Fig. 8A). Consistently, intracellular Fe^2+^ levels were also increased under the same conditions (Fig. 8B). Western blot analysis further showed that combined ALOX15 inhibitor and LPS treatment elevated ALOX15 protein levels while reducing GPX4 expression (Fig. 8C), collectively confirming the occurrence of ferroptosis in these cells.

Notably, ALOX15 protein levels were elevated in LPS-treated MCECs even in the presence of the ALOX15 inhibitor, suggesting compensatory up-regulation under inflammatory conditions. Importantly, cotreatment with RA markedly reversed lipid peroxidation, Fe^2+^ accumulation, and ferroptosis-related protein expression changes induced by PD146176 and LPS (Fig. 8A to C). Immunofluorescence staining and mitochondrial membrane potential (MMP) assays showed consistent trends (Fig. 8D and E), further supporting these findings. Taken together, these results identify ALOX15 as a critical mediator of RA’s protective action against LPS-induced ferroptosis in MCECs, with RA exerting its effect through inhibitory modulation of ALOX15 activity.

### Proteomic analysis of RA-treated MCECs

**Fig. 9.**
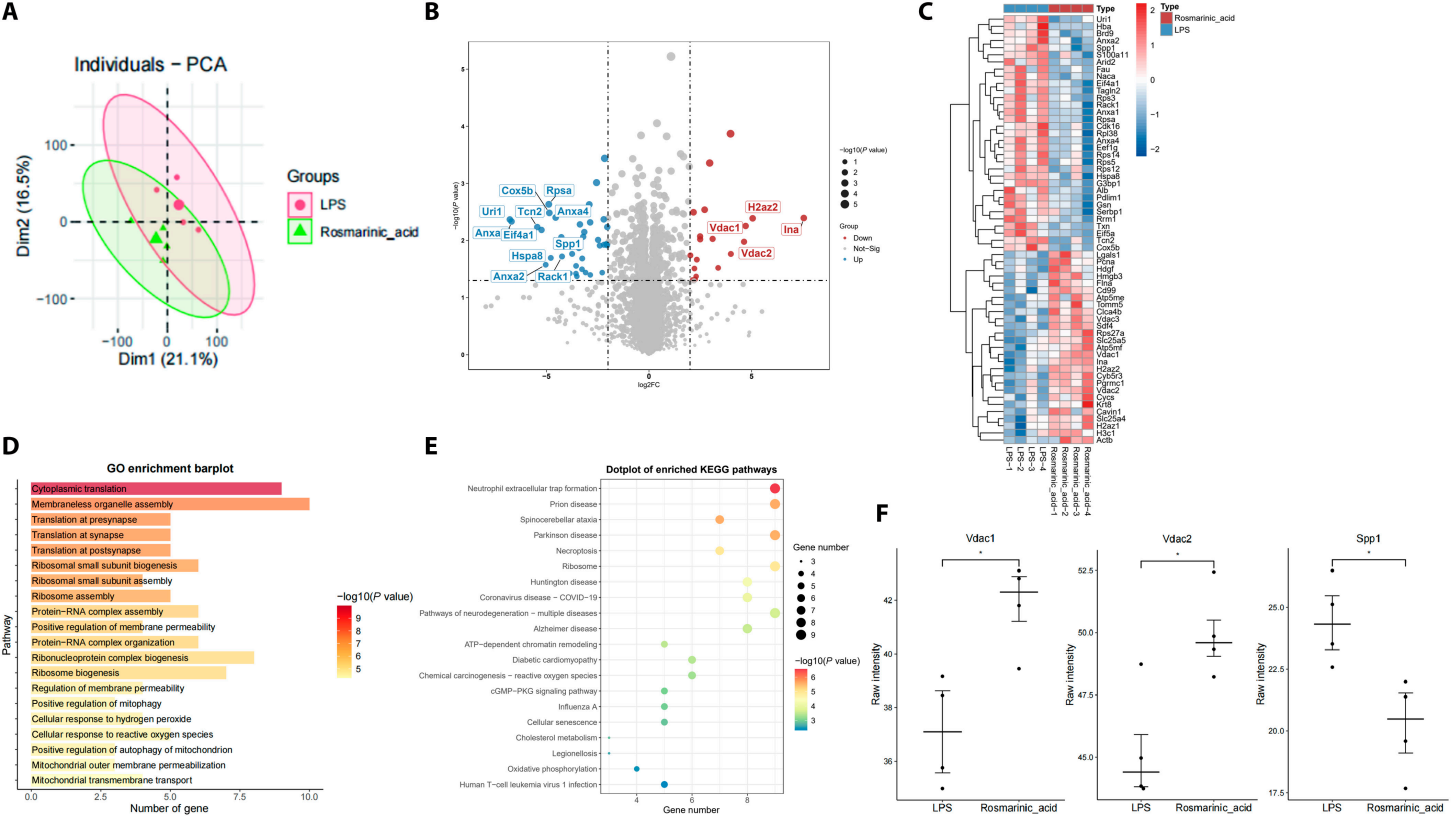
The effect of rosmarinic acid on ferroptosis-related protein spectrum proteomic analysis of rosmarinic acid-induced changes in ferroptosis-related proteins. (A) PCA plot comparing protein expression profiles between RA-treated and LPS-treated groups. (B) Volcano plot highlighting proteins with differential expression between the LPS and RA groups. (C) Heatmap displaying the 30 most variably expressed proteins when comparing rosmarinic acid and LPS treatment conditions. (D) Bar graph of the most markedly enriched GO biological terms identified among proteins with altered expression. (E) The 20 highest-ranking KEGG pathways associated with differentially expressed proteins. (F) Individual proteins showing notable expression changes in response to rosmarinic acid.

To characterize overall protein expression differences between sample groups, principal component analysis (PCA) revealed clear separation between the RA treatment group and the LPS group (Fig. 9A). To further investigate the mechanistic basis of these proteomic changes, we compared protein expression profiles in RA-treated and untreated MCECs. Using thresholds of *P* < 0.05 and |log₂(fold change)| > 2, a total of 65 differentially expressed proteins (DEPs) were identified (Fig. 9B). Hierarchical clustering analysis displayed in a heatmap revealed substantial differences in the top 30 DEPs between the LPS and RA treatment groups (Fig. 9C).

Following RA treatment, Gene Ontology (GO) analysis showed that the most notably enriched biological processes included cytoplasmic translation, cellular response to ROS, and positive regulation of mitophagy (Fig. 9D), suggesting a functional link between RA treatment and modulation of oxidative stress and protein homeostasis. Notably, KEGG pathway analysis showed that DEPs were markedly enriched in neutrophil extracellular trap formation, necroptosis, and oxidative phosphorylation pathways (Fig. 9E), indicating a potential regulatory role of RA in ferroptosis in UC. Furthermore, marked expression changes were observed in several proteins associated with ROS suppression following treatment (Fig. 9F).

### VDAC1 KD and ALOX15 overexpression rescue assay verify the key role of the ALOX15–VDAC1 axis

To further confirm that RA protects against mitochondrial dysfunction and ferroptosis via the ALOX15–VDAC1 axis, VDAC1 KD and ALOX15 overexpression rescue assays were performed in MCECs.

**Fig. 10.**
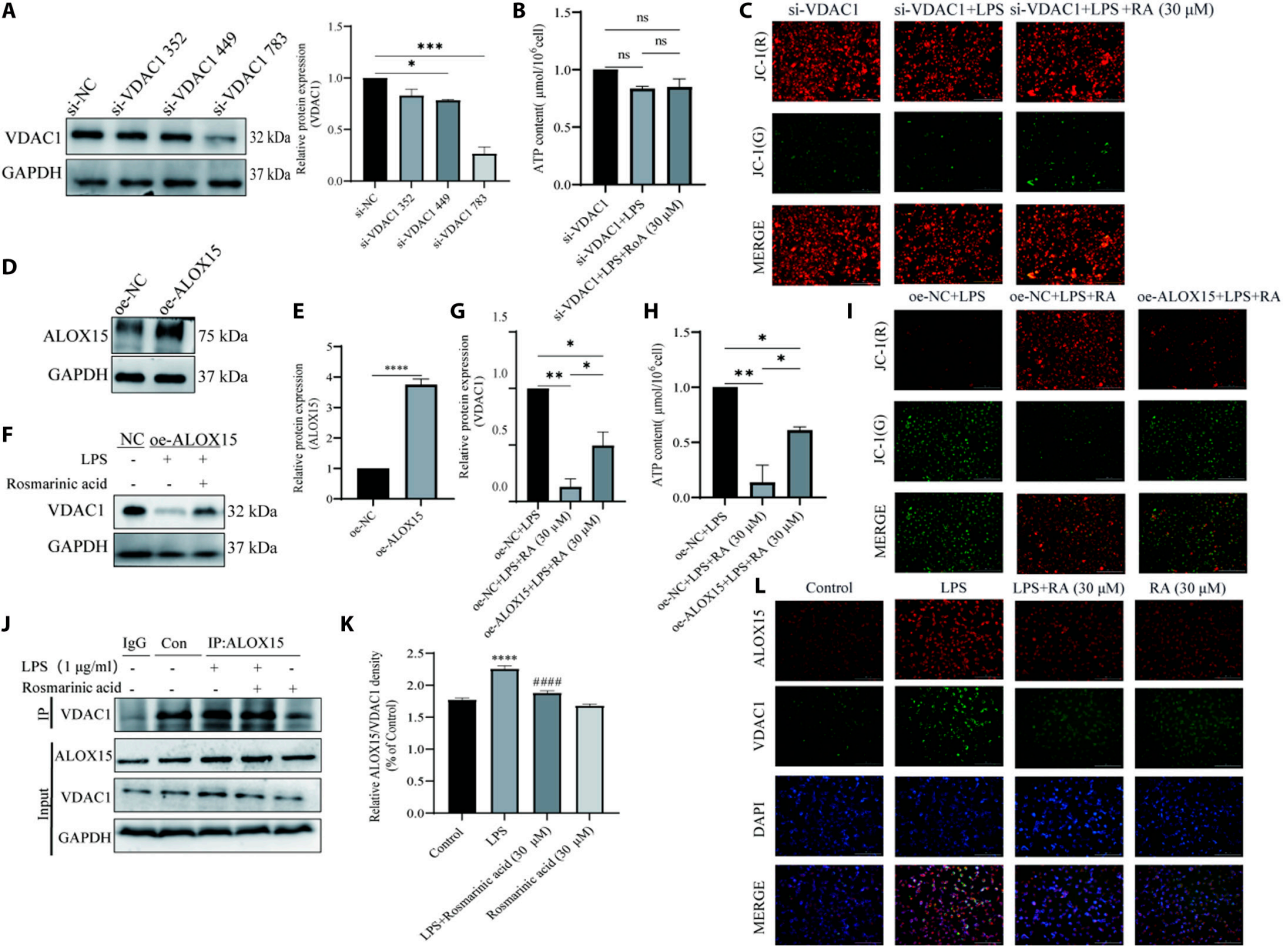
VDAC1 knockdown and ALOX15 overexpression verify that RA protects against mitochondrial dysfunction via the ALOX15–VDAC1 axis. (A) Western blot validation of VDAC1 knockdown efficiency in MCECs. (B) Quantification of intracellular ATP levels in VDAC1-knockdown MCECs under indicated treatments.(C) Representative JC-1 staining images to detect mitochondrial membrane potential in VDAC1 knockdown groups; red fluorescence represents intact mitochondrial potential, green fluorescence indicates mitochondrial depolarization. (D) Western blot and corresponding quantitative histogram confirming ALOX15 overexpression efficiency in MCECs. (E) Quantitative histogram for relative ALOX15 expression from panel (D). (F) Western blot of VDAC1 expression in ALOX15 overexpression rescue experiment. (G) Quantitative statistics of VDAC1 protein derived from panel (F). (H) Intracellular ATP quantification in ALOX15 overexpression rescue groups. (I) JC-1 fluorescence images showing mitochondrial potential in oe-ALOX15 groups. (J) Co-IP Western blot assay detecting endogenous physical interaction between ALOX15 and VDAC1; IgG served as negative control; Input denotes total cellular protein loading of each group. (K) Quantitative gray-scale statistical column diagram of Co-IP pulled-down VDAC1/ALOX15 relative density, normalized to the Control group. (L) Representative confocal double immunofluorescence staining of ALOX15 (red) and VDAC1 (green), DAPI (blue) for nuclear counterstaining across 4 groups (Control, LPS, LPS+RA, and RA alone); scale bar = 100 μm. Data are expressed as mean ± SD. *P < 0.05, **P < 0.01, ***P < 0.001 vs. control group; #*P* < 0.05, ##*P* < 0.01 vs. LPS group; &*P* < 0.05, &&*P* < 0.01 vs. LPS+RA group.

Western blot confirmed that VDAC1 was efficiently knocked down (Fig. 10A). In VDAC1-KD cells, LPS-induced MMP collapse and adenosine triphosphate (ATP) reduction were markedly alleviated, and RA could not further enhance these protective effects (Fig. 10B and C), indicating that VDAC1 is an essential downstream mediator of RA-mediated mitochondrial protection.

In the ALOX15 overexpression rescue assay, ALOX15 overexpression markedly reversed RA-induced VDAC1 down-regulation (Fig. 10D). Meanwhile, the recovery of MMP and elevation of ATP levels by RA were also abolished by ALOX15 overexpression (Fig. 10E and F).

To further verify whether RA regulates the ALOX15–VDAC1 axis by interfering with the endogenous protein–protein interaction between ALOX15 and VDAC1, co-immunoprecipitation (Co-IP) and immunofluorescence colocalization assays were performed in MCECs. Co-IP results revealed that LPS stimulation markedly enhanced the physical binding between endogenous ALOX15 and VDAC1, whereas RA pretreatment substantially reduced their coprecipitation levels. Consistently, immunofluorescence double staining combined with confocal microscopy showed elevated colocalization of ALOX15 and VDAC1 upon LPS stimulation. RA administration effectively decreased overlapping fluorescent signals, confirming that RA impairs formation of the ALOX15–VDAC1 protein complex. These results demonstrate that RA inhibits ferroptosis and enhances mitochondrial function in intestinal epithelial cells by regulating the ALOX15–VDAC1 signaling axis.

### RA and Mito-TEMPO alleviate LPS-induced mitochondrial dysfunction associated with ferroptosis

**Fig. 11.**
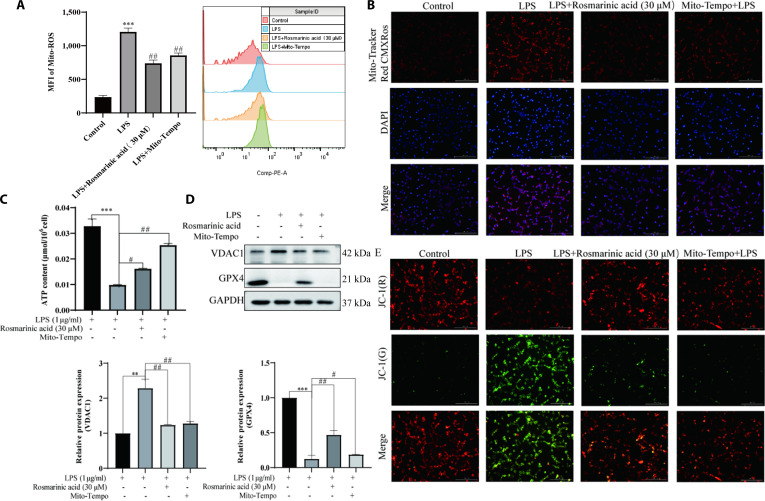
Rosmarinic acid prevents mitochondrial dysfunction following LPS-induced cell damage by inhibiting the up-regulation of VDAC1. (A) Mitochondrial ROS levels detected using a Mito-ROS probe and flow cytometry. (B) Mitochondrial morphology assessed by MitoTracker Red staining (scale bar: 100 μm). (C) Intracellular ATP levels. (D) Western blot analysis of GPX4 and VDAC1 protein expression in mitochondria. (E) Mitochondrial membrane potential assessed by JC-1 staining (scale bar: 100 μm). Data are expressed as mean ± SD; *P < 0.05, **P < 0.01, ***P < 0.001 compared with the control group; #*P* < 0.05, ##*P* < 0.01, ###*P* < 0.001 compared with the LPS-treated group.

Previous studies have shown that VDAC1 promotes mitochondrial dysfunction during ferroptosis. Therefore, we sought to determine whether VDAC1 is a key component of this inflammatory injury model. We first evaluated the effect of RA on ATP generation. As shown in Fig. 11C, ATP levels were markedly reduced in LPS-treated cells compared with the control group. Given that MMP decline occurs at an early stage of mitochondrial damage, MMP was assessed using 5,5′,6,6′-tetrachloro-1,1′,3,3′-tetraethylbenzimidazolylcarbocyanine iodide (JC-1) staining. Compared with the control group, LPS treatment led to a marked reduction in the red/green fluorescence ratio (Fig. 11E), indicating mitochondrial depolarization.

Mitochondria are widely recognized as the primary source of intracellular ROS. Using the Mito-ROS assay, mitochondrial ROS levels were found to increase sharply following LPS treatment, an effect markedly counteracted by RA (Fig. 11A). Mito-TEMPO is a mitochondria-targeted superoxide dismutase mimetic that accumulates within the inner mitochondrial membrane via triphenylphosphonium-mediated uptake, thereby reducing LPS-induced mitochondrial ROS accumulation. Consistent with these findings, mitochondrial GPX4 protein expression was progressively reduced in LPS-damaged cells. Western blot analysis revealed that VDAC1 protein levels were markedly up-regulated in damaged cells, and both RA and Mito-TEMPO treatment partially reversed these changes (Fig. 11D). MitoTracker Red CMXRos staining further supported these observations (Fig. 11B). Taken together, these results demonstrate that both RA and Mito-TEMPO promote VDAC1 and its oligomer degradation, thereby contributing to mitochondrial homeostasis.

## Discussion

This study delineates a coherent mechanistic pathway through which RA alleviates UC. The ALOX15–VDAC1 axis emerges as a promising therapeutic target through which RA exerts its effects via ferroptosis suppression. This multidisciplinary work, integrating bioinformatics discovery, artificial intelligence-driven screening, and rigorous experimental validation, provides a framework linking target identification to functional outcomes.

Our investigation was motivated by the unmet clinical need in UC and the emerging pathogenic role of ferroptosis. Bioinformatics analysis of patient-derived data identified ALOX15 as a markedly up-regulated ferroptosis-related gene in UC with important diagnostic potential [2,5]. This finding provides a robust clinical and molecular rationale for targeting ALOX15 as a central therapeutic node, supporting a causal role in disease progression.

To identify compounds targeting this pathway, we employed a robust computational strategy. A machine learning model prioritized RA from a natural compound library [32–43], consistent with and extending our previously established protocol for ALOX15 inhibitor discovery [44]. Molecular docking and MD simulations structurally validated this prediction, revealing a stable and high-affinity binding mode in the active site of ALOX15. Critically, this predicted interaction was experimentally confirmed under physiological conditions via CETSA, DARTS, and Co-IP assays, demonstrating that their interaction improves the stability of ALOX15 protein. This integrated pipeline, from in silico prediction to biophysical and cellular validation, represents a robust framework for natural product-based drug discovery and establishes ALOX15 as a direct and actionable molecular target of RA.

Following candidate selection by the machine learning model, we experimentally confirmed that RA effectively inhibits ferroptosis and protects mitochondrial function in vitro and in vivo [45].

Having confirmed target engagement, we demonstrated the functional consequences of this interaction. RA inhibited ferroptosis in a concentration-dependent manner in vitro, restoring core anti-ferroptotic regulators (GPX4 and SLC7A11) and suppressing pro-ferroptotic drivers (ACSL4 and COX-2) [7,8,17]. Crucially, ALOX15 KD abolished the protective effects of RA, providing clear genetic evidence that ALOX15 is essential for RA-mediated cytoprotection, establishing a mechanistic relationship rather than a simple correlation. The in vivo relevance of this axis was further confirmed. RA ameliorated DSS-induced colitis by up-regulating tight junction proteins (ZO-1, Occludin, and Claudin-1), reducing disease activity scores, and restoring the integrity of the intestinal barrier. By preventing ferroptosis in epithelial cells [19], RA preserves the epithelial barrier, which represents a central component of UC pathogenesis.

Our research further elucidates the key downstream pathways that connect ALOX15 inhibition and mitochondrial homeostasis. Consistent with the proteomic findings, serum metabolomic analysis demonstrated that RA extensively remodels lipid metabolism pathways (including arachidonic acid metabolism, fatty acid oxidation, and linolenic acid metabolism), thereby linking its therapeutic effects to reduced lipid peroxidation and suppressed ferroptosis in colitis. Proteomic analysis and subsequent verification demonstrated that RA-mediated ALOX15 inhibition leads to VDAC1 down-regulation. As a mitochondrial gatekeeper, VDAC1 dysregulation is a known factor in ferroptotic damage [9,42]. Our findings demonstrate that RA treatment effectively reversed the up-regulation of VDAC1, while preserving MMP, suppressing mitochondrial ROS production, and restoring ATP levels. Furthermore, we provide direct genetic evidence through VDAC1 KD and ALOX15 overexpression rescue assays: VDAC1 KD abolished the mitochondrial protective effect of RA, while ALOX15 overexpression reversed RA-mediated regulation of VDAC1 and mitochondrial function, thereby confirming that the ALOX15–VDAC1 axis is an indispensable pathway for the anti-UC effect of RA. These findings support a mechanistic model in which upstream inhibition of ALOX15-driven lipid peroxidation reduces the direct damage to the mitochondrial membrane, thus preventing VDAC1 dysregulation and interrupting ROS-induced mitochondrial damage and further ROS production and metabolic collapse. The comparable effects of RA and the mitochondria-targeted antioxidant Mito-TEMPO further underscore the central importance of mitochondrial recovery in the mechanism of action of RA.

Further Co-IP and confocal colocalization data with quantitative Pearson coefficient calculation demonstrated that LPS promotes endogenous ALOX15–VDAC1 complex formation, while RA disrupts their physical interaction to down-regulate VDAC1 expression and ameliorate mitochondrial dysfunction, further consolidating the regulatory role of RA within the ALOX15–VDAC1 axis.

**Fig. 12.**
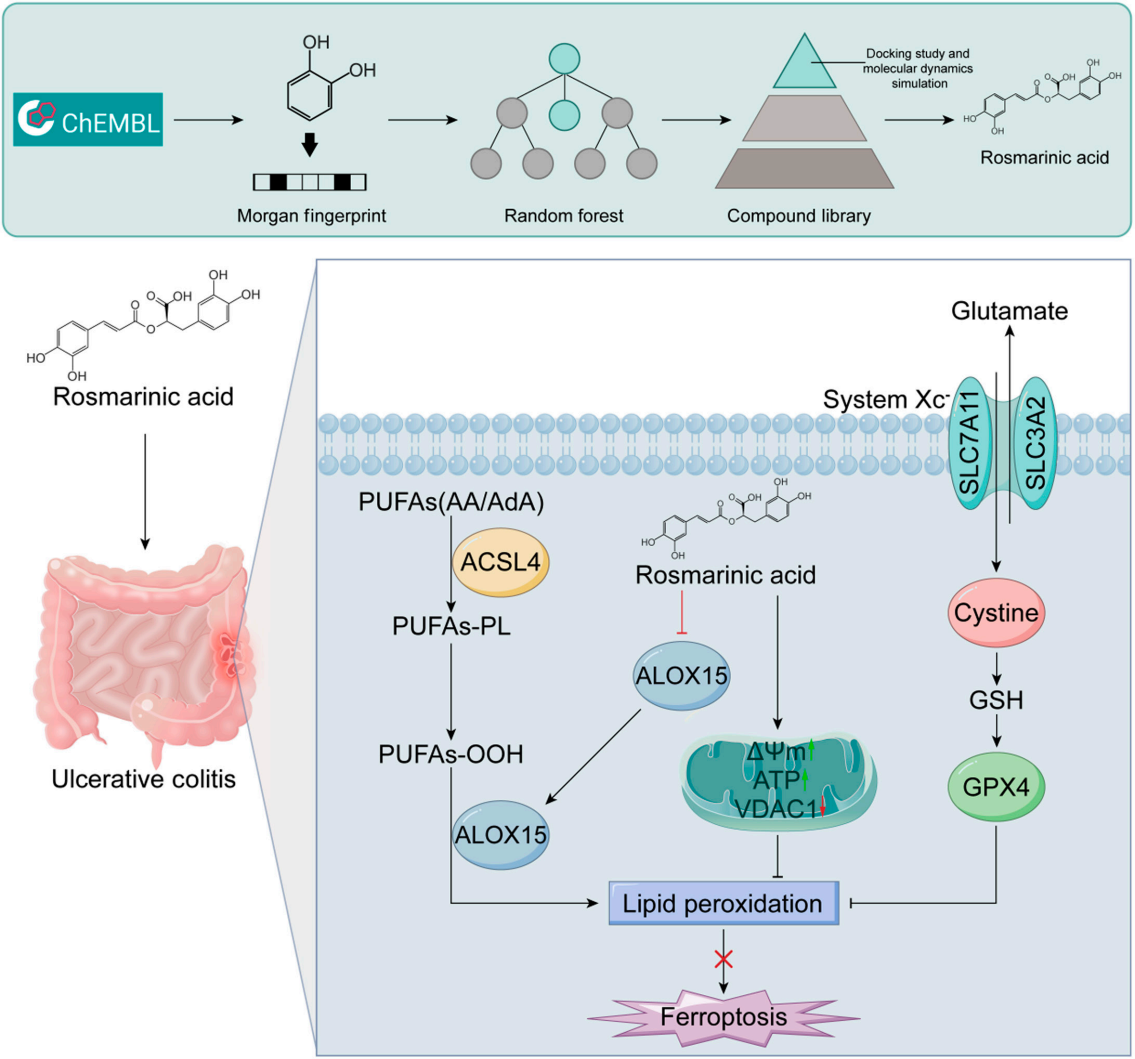
Schematic diagram of the molecular mechanism proposed in this study. ACSL4, acyl-CoA synthetase long-chain family member 4; ALOX15, arachidonate 15-lipoxygenase; System Xc^−^, cystine/glutamate antiporter system (composed of SLC7A11 and SLC3A2 subunits); SLC7A11, solute carrier family 7 member 11; SLC3A2, solute carrier family 3 member 2; GSH, glutathione; GPX4, glutathione peroxidase 4; PUFAs, polyunsaturated fatty acids (including AA/AdA); PUFAs-PL, phospholipid-bound polyunsaturated fatty acids; PUFAs-OOH, peroxidized polyunsaturated fatty acids. Solid black arrows (→) indicate promoting or activating effects in the ferroptosis pathway. Red inhibitory arrow (⊥) represents the direct inhibitory effect of rosmarinic acid on ALOX15 activity. Red cross (×) indicates the blockade of ferroptosis progression by rosmarinic acid-mediated suppression of lipid peroxidation. Transmembrane proteins (SLC7A11/SLC3A2) represent the System Xc^−^ antiporter, which mediates cystine uptake and glutamate efflux. The left schematic depicts the therapeutic targeting of rosmarinic acid to the inflamed colon tissue in ulcerative colitis. This schematic illustrates that rosmarinic acid exerts therapeutic effects in ulcerative colitis by functional inhibition in ALOX15, thereby reducing the peroxidation of membrane phospholipid-bound polyunsaturated fatty acids (PUFAs-PL) and subsequent lipid peroxidation cascades. This blockade ultimately suppresses ferroptosis in intestinal epithelial cells. The System Xc^−^–GSH–GPX4 axis is the canonical negative regulatory pathway of ferroptosis, where GPX4 eliminates lipid peroxides to prevent ferroptotic cell death.

In summary, this study identifies the previously uncharacterized RA–ALOX15–VDAC1 axis as a mechanistic basis for RA’s therapeutic effects in UC (Fig. 12). Our work extends the traditional view of RA as a broad-spectrum antioxidant, repositioning it as a selective inhibitor of specific pro-ferroptotic enzymes. This discovery adds a new dimension to the growing body of evidence that natural products can regulate ferroptosis in human disease. Despite these findings, several limitations merit acknowledgement. Notably, this study lacks an independent RA-alone control group in in vivo experiments. Given the well-documented biosafety of RA, no appreciable histological injury or inflammatory abnormality was observed in healthy mice administered moderate doses of RA in previous studies. Nevertheless, the absence of a single RA-treated group for blank comparison is an undeniable limitation, and further research will supplement this group to clarify the direct biological effect of RA on normal intestinal tissue. The use of gene KD models has inherent limitations; future research using the inducible cell-specific knockout model will provide more accurate insights. The translational potential of RA also needs to be evaluated in human-relevant systems, including patient-derived tissues and organoid models, such as intestinal organoid models. The promising preclinical efficacy of RA necessitates a critical evaluation of its translational pathway. Pharmacokinetically, RA exhibits moderate oral bioavailability, with its glycosidic derivatives and colonic microbiota metabolism potentially enhancing its local concentration in the gut—a property potentially advantageous for UC treatment [46]^.^ Its established safety profile, supported by a long history of dietary consumption and a high LD₅₀ in rodents [47–49], positions it favorably for further development, possibly as a nutraceutical or dietary adjunct. However, key challenges must be addressed to advance its therapeutic application. These include optimization of formulation strategies to improve its stability and targeted colonic delivery, rigorous long-term toxicity evaluation, and determination of whether the effective concentrations achieved in vitro and in murine models are translatable to human pathophysiology. Future work should prioritize validation in human intestinal organoids or primary epithelial cells from UC patients, and explore potential synergies with current first-line therapies such as mesalazine. Finally, future research should explore the crosstalk between RA-regulated ferroptosis and other cell death pathways.

In addition, several limitations related to the machine learning-based virtual screening framework should be acknowledged. Although a strict scaffold-disjoint validation strategy was employed to minimize optimistic bias and prevent structural leakage, the independent holdout subset ultimately contained a relatively limited number of Murcko scaffold series due to the highly imbalanced scaffold-size distribution of the curated ChEMBL dataset. Broader scaffold-level generalization was therefore primarily assessed through repeated scaffold-grouped OOF validation across the train-development subset rather than by the independent holdout evaluation alone. Moreover, the machine learning framework presented in this study should be interpreted as a candidate-prioritization and virtual-screening tool rather than definitive mechanistic evidence. While the model demonstrated stable discriminative performance under scaffold-disjoint evaluation settings, computational predictions alone cannot establish direct biochemical causality or guarantee generalization to entirely unseen chemical spaces. Additional large-scale datasets, external validation cohorts, and prospective experimental evaluations will be necessary to further improve the robustness and translational applicability of the screening framework. Despite these future directions, our findings highlight that the ALOX15–VDAC1 axis represents a promising therapeutic target for future drug development in inflammatory bowel disease.

We demonstrated that RA, identified via machine learning as an ALOX15 inhibitor, alleviates UC by modulating ALOX15 expression, down-regulating VDAC1, and thereby inhibiting VDAC1-mediated ferroptosis and mitochondrial dysfunction. Metabolomics profiling further demonstrated that RA regulates lipid metabolism and oxidative stress to alleviate colitis. Additionally, RA was confirmed to suppress ALOX15–VDAC1 complex formation, thereby blocking downstream mitochondrial damage and ferroptosis in intestinal epithelial cells. These results highlight the therapeutic potential of RA and identify the ALOX15–VDAC1 pathway as a promising target for the treatment of inflammatory bowel disease.

## Materials and Methods

### Machine learning-based virtual screening

ALOX15 inhibitor records were collected from ChEMBL (*n* = 904). Molecular structures were standardized and validated using RDKit, and invalid SMILES were excluded. Duplicate molecular entries were aggregated by mean activity values, yielding 698 unique compounds for downstream modeling. IC_50_ values were converted to pIC_50_, and binary labels were assigned using pIC_50_ ≥ 5.2 as the activity threshold. Features were generated from Morgan fingerprints, MACCS fingerprints, and pretrained molecular representations. The pretrained representation was constructed using approximately 3 million drug-like molecules collected from the ZINC database without using any ALOX15 activity annotations. The pipeline proceeded as follows: Morgan fingerprints (radius = 2, 2,048 bits) and MACCS keys (167 bits) were computed for each pretraining molecule and concatenated into an initial feature vector; missing values were imputed using the median strategy; columns with variance below 0.16 were discarded; features were standardized to zero mean and unit variance using StandardScaler; and finally, IPCA was performed with a batch size of 4,096 to reduce the feature dimensionality to 128. The fitted imputer, variance selector, scaler, and IPCA projector were serialized as the pretraining preprocessor artifact.

During downstream ALOX15 classification modeling, each molecule’s Morgan and MACCS fingerprints were first computed, then passed through the imputation, filtering, and standardization steps of the pretrained preprocessor, and finally projected via the frozen IPCA model to yield a 128-dimensional pretrained representation. The final model input was formed by concatenating the filtered raw fingerprints with this 128-dimensional representation. The rationale for this hybrid feature design is twofold: Morgan and MACCS fingerprints capture local substructural and functional group information directly relevant to target-specific activity, whereas the IPCA-reduced representation encodes the global manifold structure of the chemical space learned from large-scale data, providing informative priors for scaffold regions underrepresented in the training set. Their combination is particularly advantageous for improving generalization under strict scaffold extrapolation.

To ensure rigorous generalization assessment and minimize optimistic bias caused by structural similarity, a strict scaffold-disjoint validation framework was adopted. Molecules were grouped according to Bemis–Murcko scaffolds, followed by scaffold-level train-development/test partitioning (9:1), in which scaffold groups were greedily assigned to the independent test subset until the predefined test size threshold was reached, ensuring zero scaffold overlap between the training and independent test subsets. Within the train-development subset, 5-fold scaffold GroupKFold cross-validation was further applied, such that molecules sharing the same scaffold were always assigned to the same fold. The detailed scaffold distribution across outer validation folds is summarized in Table S4. During benchmark comparison, all candidate classifiers were evaluated under the identical scaffold partitioning and outer-fold cross-validation protocol. For the finalized ExtraTrees framework, hyperparameter optimization was performed exclusively within nested inner-fold cross-validation restricted to each outer training subset, whereas outer validation folds and the independent scaffold-disjoint test set were reserved strictly for unbiased performance estimation and never participated in model selection, threshold optimization, or feature engineering. The optimal hyperparameters identified across outer folds are summarized in Table S7. The final classifier deployed for holdout testing and virtual screening was a 5-fold scaffold ensemble, formed by averaging positive-class probabilities from the 5 outer-fold ExtraTrees models trained under nested cross-validation.

Model performance was evaluated using both scaffold-grouped OOF predictions and an independent scaffold-disjoint test set, including ROC-AUC, PR-AUC, F1 score, balanced accuracy, precision, recall, and MCC. Decision thresholds were determined exclusively from OOF predictions and subsequently applied unchanged to the independent test set, thereby avoiding threshold-selection bias during holdout evaluation. To quantify statistical uncertainty, stratified bootstrap resampling (2,000 iterations) was further applied to estimate 95% CIs for ROC-AUC and PR-AUC. Collectively, this validation framework ensured that structurally related molecules were not simultaneously exposed across training, validation, and independent test subsets, thereby minimizing potential information leakage during model development and evaluation. In addition, Y-randomization analysis (499 label permutations) was performed to evaluate the likelihood of chance correlations. After model comparison, ExtraTrees was selected as the final classifier and used for prospective virtual screening of an external candidate library, followed by docking-based prioritization and experimental follow-up.

### Transcriptomic data acquisition and processing

The bulk RNA-seq dataset GSE117993, related to pediatric UC, was retrieved from the Gene Expression Omnibus (GEO) database, comprising 55 healthy control and 43 UC patient samples. To ensure analytical rigor, only UC and control samples were retained; Crohn’s disease samples were excluded. Duplicate entries were removed. For each sample, the GEO accession, GEO Platform (GPL), and disease status were recorded.

### Identification of DEGs and diagnostic evaluation

DEGs associated with UC were identified from the merged expression matrix using the R limma package, with significance cutoffs of false discovery rate (FDR) < 0.05 and |log₂FC| > 1. The diagnostic capacity of ALOX15 was evaluated through ROC curve analysis and AUC calculation employing the *pROC* package.

### Functional enrichment methodology

Using the median expression of ALOX15 as a cutoff, samples were categorized into high- and low-expression groups. Differential enrichment across 114 metabolic pathways was assessed by integrating the GSVA algorithm with the *limma* package (|*t*| > 0 defined as the significance criterion). Simultaneously, GSEA based on KEGG pathways was applied to DEGs (*P* < 0.05) using clusterProfiler (*P* < 0.05).

### Proteomic profiling and annotation

Proteomics data were processed using DIA-NN (v1.8.1) with a library-free search against UniProtKB, with quantification via the MaxLFQ algorithm. The *limma* package facilitated DEP identification (*P* < 0.05, |log₂FC| > 2). Functional enrichment (GO/KEGG) was performed with *clusterProfiler*, and protein feature diagrams were generated using *drawProteins* and *ggplot2*.

### Metabolomics analysis

Serum metabolic profile data were obtained from C57 mice. The metabolic data were log-transformed and mean-centered using the SIMCA software (V16.0.2), followed by PCA and orthogonal partial least squares discriminant analysis. Metabolites with *P* < 0.05 and a VIP greater than 1 were selected as differential metabolites. Visualization was performed using R (v4.5.0). Subsequently, the differential metabolites were subjected to KEGG and SMPDB enrichment analysis through MetaboAnalyst.

### In vivo experimental design

C57BL/6 mice (*n* = 5 per group) were acclimated for 2 weeks prior to random allocation into 3 groups: a control group, a DSS group (DSS sourced from MP Biomedicals, UK), and a DSS+RA group (RA obtained from Shaanxi Tianduoli Biological Technology Co., Ltd., China). The administered dose of RA (100 mg/kg) was determined based on previously published pharmacological studies focusing on RA. This moderate dose within the commonly used effective range (50 to 150 mg/kg) was selected to ensure stable anti-inflammatory and antioxidant activity with negligible systemic toxicity, consistent with prior in vivo investigations of RA in murine inflammatory models [29,50,51]. The DSS+RA group received daily intragastric administration of RA (100 mg/kg) throughout the experimental period (days 0 to 10). Colitis was induced in both the DSS and DSS+RA groups by providing 3% (w/v) DSS in drinking water from day 3 to day 10. Body weight and DAI were recorded daily. On day 11, mice were euthanized, blood was collected via the retro-orbital plexus, and colons were harvested. Colon segments were either fixed in 4% paraformaldehyde (PFA) or snap-frozen. Serum, prepared by centrifuging blood at 12,000 rpm for 10 min (4 °C), was aliquoted and stored at −80 °C.

### Histological and immunohistochemical analysis

H&E staining was performed on colon tissues fixed in 4% PFA (Macklin, China). Subsequent processing included paraffin embedding, sectioning, deparaffinization, rehydration and staining according to standard protocols.

For immunohistochemistry (IHC) staining, tissue sections were deparaffinized, rehydrated, and subjected to heat-mediated antigen retrieval. To block endogenous peroxidase activity, sections were incubated with 3% H₂O₂ (Nanyue, China) for 15 min. Nonspecific binding was minimized by incubating sections in 3% goat serum (Sangon Biotech, China) for 30 min at room temperature. Primary antibodies targeting ZO-1, Occludin, Claudin-1, MUC-2, and ALOX15 (ABclonal, China), diluted in 5% bovine serum albumin (BSA), were then applied overnight at 4 °C. Following thorough washing, horseradish peroxidase (HRP)-conjugated secondary antibodies (1:5,000; CST, China) were applied for 30 min. Color development was carried out with DAB solution, nuclei were counterstained with hematoxylin, and coverslips were mounted using neutral resin (Solarbio, China).

### Cell culture and treatments

The mouse colon epithelial cell line MCEC and rat intestinal epithelial cell line IEC-6 (Fenghui Biotechnology Co., Ltd., China) were cultured in Dulbecco's Modified Eagle Medium (Gibco, USA) supplemented with 10% fetal bovine serum at 37 °C under 5% CO₂. For experiments, cells were seeded in 6-well plates (3 × 10^6^ cells/well) and treated for 24 h with RA (0, 10, 20, or 30 μM) or LPS (1 μg/ml; Sigma-Aldrich, USA). The LPS+RA group received 2 h of RA pretreatment prior to LPS exposure. For ALOX15 inhibition experiments, cells were pretreated with PD146176 (10 μM) for 1 h before LPS stimulation.

### Cell viability assay (CCK-8)

MCECs were seeded in 96-well plates (4 × 10^3^ cells/well). Following the treatment period, 10 μl of CCK-8 reagent (NCM Biotech, China) was added per well and incubated for 3 h. Absorbance at 450 nm was measured to determine cell viability.

### Intracellular iron measurement

Cells were loaded with 1 μM FerroOrange (Dojindo, Japan) in serum-free medium for 20 min at 37 °C and washed twice with phosphate-buffered saline (PBS) (Sangon Biotech, China), and fluorescence intensity was quantified using a Cytation 5 microplate reader (BioTek, USA).

### Lipid peroxidation assay

Cells were stained with 10 μM C11-BODIPY^581/591^ (ABclonal, China) for 30 min at 37 °C, washed with PBS, and analyzed by flow cytometry (DXP Athena, Cytek Biosciences, USA).

### Measurement of GSH and MDA

Intracellular GSH levels were quantified using a DTNB colorimetric assay kit (Beyotime, cat. no. S0053), and MDA content was measured via a thiobarbituric acid method kit (Beyotime, S0131S), strictly following the manufacturer’s protocols.

### Western blotting

Cells were lysed in radioimmunoprecipitation assay buffer supplemented with protease inhibitors and phenylmethylsulfonyl fluoride (Solarbio, China). After protein quantification, equal amounts of lysate were separated by SDS-PAGE (Sangon Biotech, China) and transferred to nitrocellulose membranes. Membranes were blocked with 5% BSA (Solarbio, China) for 30 to 60 min, then probed overnight at 4 °C with primary antibodies against GPX4, FTH1, SLC7A11 (CST, China), COX-2, ACSL4, ALOX15, Occludin, Claudin-1, and ZO-1 (ABclonal, China). After incubation with HRP-conjugated secondary antibodies (1:5,000; CST, China), bands were visualized using BeyoECL Moon reagent (Beyotime Biotech, China). GAPDH and β-actin served as loading controls.

### Cellular thermal shift assay

MCEC suspensions were subjected to a thermal gradient (40 to 80 °C, 10 min), chilled on ice (5 min), and centrifuged (12,000×*g*, 10 min, 4 °C). The resulting supernatants were subjected to Western blot analysis.

### DARTS assay

MCEC lysates were incubated with RA for 1 h at room temperature, followed by proteolytic digestion with Pronase (Roche, Switzerland) at an enzyme-to-protein ratio of 1:300 for 40 min at 37 °C. Reactions were terminated by boiling for 3 min prior to Western blot analysis.

### Immunofluorescence staining

Following fixation (4% formaldehyde) and permeabilization (0.5% Triton X-100), cells were incubated with primary antibodies at 4 °C overnight. After washing, fluorescent secondary antibodies and 4′,6-diamidino-2-phenylindole (DAPI) were applied for signal detection and nuclear counterstaining, respectively. Images were acquired using an FV3000 confocal microscope (Olympus, Japan).

### Co-immunoprecipitation

Cell lysates were incubated with an anti-ALOX15 antibody. The immunoprecipitated complex was collected, washed, and analyzed by Western blot.

### MMP assay

MMP was assessed using JC-1 staining according to the manufacturer’s instructions, with subsequent imaging by fluorescence microscopy.

### Molecular docking

Compound libraries were prepared using LigPrep (Schrödinger) at pH 7.0 ± 2.0. The ALOX15 protein structure (AlphaFold ID: P16050) was prepared using the Protein Preparation Wizard. Docking was performed in extra-precision mode with a defined receptor grid (center coordinates: 4.03, 6.16, 4.3 Å; grid dimensions: 36 Å) enclosing the active site.

### MD simulation

The ALOX15–RA complex was subjected to a 100-ns MD simulation using the AMBER99SB-ILDN force field. Ligand parameters were generated with Sobtop/GAFF. The system was solvated, neutralized, energy-minimized, and equilibrated before the production run. Trajectories were analyzed for RMSD, RMSF, Rg, and FEL.

### VDAC1 KD assay

MCECs were transfected with VDAC1 siRNA (5′-UAAAUUCCAAUCCAUUCUC-3′) or negative control siRNA for 48 h. The KD efficiency was verified by Western blot. Transfected cells were allocated into LPS (1 μg/ml, 24 h) and LPS + RA (30 μM, 2 h pretreatment) groups, and MMP and ATP levels subsequently measured.

### ALOX15 overexpression rescue assay

MCECs were transfected with ALOX15 overexpression plasmid or empty vector for 48 h. Overexpression efficiency was confirmed by Western blot. Cells were allocated into empty vector + LPS, empty vector + LPS + RA, and ALOX15 overexpression + LPS + RA groups. LPS (1 μg/ml) and RA (30 μM) were used for 24 h treatment with 2 h pretreatment. VDAC1 expression, MMP, and ATP levels were measured.

### Statistical analysis

All quantitative data are expressed as mean ± standard deviation (SD). Statistical analyses were performed using GraphPad Prism (version 9.5). The normality of data distribution was assessed using the Shapiro–Wilk test. For comparisons between 2 groups, an unpaired 2-tailed Student *t* test was applied. For comparisons among 3 or more groups, one-way analysis of variance (ANOVA) followed by Tukey’s post hoc test for multiple comparisons was used. For data involving repeated measurements over time (e.g., body weight and disease activity index), 2-way repeated-measures ANOVA followed by Šidák’s multiple comparisons test was employed. A *P* value < 0.05 was considered statistically significant. The specific statistical tests used for each dataset are detailed in the corresponding figure legends.

## Ethical Approval

All animal procedures were reviewed and approved by the Institutional Animal Care and Use Committee of Guangdong Medical University (approval no. GDY2502901). Experiments involving animals were carried out in strict accordance with national regulations and the guidelines established by the laboratory animal center at Guangdong Medical University.

## Data Availability

The datasets generated and analyzed during the current study are available from the corresponding authors on reasonable request.
