# Supplementary material for: AI-Powered Discovery of Rosmarinic Acid as a Novel Ferroptosis Inhibitor for Ulcerative Colitis via Targeting the ALOX15–VDAC1 Axis
Source: Research (Wash D C). 2026 Jul 8;9:1352. doi: 10.34133/research.1352 (PMC13342251; doi:10.34133/research.1352)
Supplement: Supplementary 1 — Tables S1 to S8 [file research.1352.f1.zip › Supplementary Information(1).docx]

Supplementary Information

Contents

- **Table S3.** Scaffold-disjoint train-development/test partition statistics
- **Table S4.** Outer 5-fold scaffold GroupKFold configuration
- **Table S5.** Fold-wise class balance across outer validation folds
- **Table S6.** Summary of leakage prevention and validation procedures
- **Table S7.** Nested cross-validation hyperparameter optimization results
- **Table S8.** Summary of Y-randomization analysis

# Supplementary Tables

## Table S3. Scaffold-disjoint train-development/test partition statistics

Summary statistics of scaffold-level dataset partitioning used for scaffold-disjoint validation.

| **Parameter** | **Value** |
| --- | --- |
| Total molecules | 698 |
| Total distinct Murcko scaffolds | 294 |
| Train-development molecules | 619 |
| Independent holdout molecules | 79 |
| Train-development scaffolds | 291 |
| Holdout scaffolds | 3 |
| Scaffold overlap between subsets | 0 |

**Note:** Due to strict scaffold-disjoint partitioning, the independent holdout subset contained a relatively limited number of Murcko scaffold series.

## Table S4. Outer 5-fold scaffold GroupKFold configuration

Configuration and scaffold distribution of the outer 5-fold scaffold GroupKFold cross-validation.

| **Parameter** | **Value** |
| --- | --- |
| Cross-validation protocol | GroupKFold (n = 5) |
| Group identifier | Murcko scaffold |
| Train-development subset size | 619 molecules |
| Distinct train-development scaffolds | 291 |
| Validation molecules per fold | 123–124 |
| Validation scaffolds per fold | 58–59 |

| **Outer fold** | **Validation molecules** | **Validation scaffolds** |
| --- | --- | --- |
| Fold 1 | 124 | 58 |
| Fold 2 | 124 | 58 |
| Fold 3 | 124 | 58 |
| Fold 4 | 124 | 59 |
| Fold 5 | 123 | 58 |

## Table S5. Fold-wise class balance across outer validation folds

Distribution of positive and negative samples across outer validation folds.

| **Outer fold** | **Validation molecules** | **Positive samples** | **Negative samples** | **Positive prevalence** |
| --- | --- | --- | --- | --- |
| Fold 1 | 124 | 70 | 54 | 56.5% |
| Fold 2 | 124 | 60 | 64 | 48.4% |
| Fold 3 | 124 | 55 | 69 | 44.4% |
| Fold 4 | 124 | 76 | 48 | 61.3% |
| Fold 5 | 123 | 53 | 70 | 43.1% |

## Table S6. Summary of leakage prevention and validation procedures

Overview of procedures used to minimize information leakage and optimistic bias during model development and evaluation.

| **Step** | **Leakage prevention strategy** |
| --- | --- |
| Independent holdout split | Test Murcko scaffolds were fully absent from the train-development subset |
| Outer cross-validation | Scaffold GroupKFold ensured each scaffold appeared in only one validation fold |
| Nested hyperparameter optimization | Inner-fold optimization was restricted to outer-fold training subsets only |
| Out-of-fold prediction | Each molecule was predicted by models that never observed that molecule during training |
| Decision threshold selection | F1-optimal threshold was determined exclusively from OOF predictions and applied unchanged to the independent test set |
| Winsorization | Quantile clipping parameters were estimated using train-development data only |
| Pretrained representation | Representation learning used external unlabeled ZINC molecules without downstream activity labels |
| Fingerprint extraction | Morgan and MACCS fingerprints were generated independently of activity labels |
| Independent test evaluation | The holdout subset was evaluated only once after model finalization |
| Virtual screening | External compounds were screened using the finalized frozen model without retraining |

## Table S7. Nested cross-validation hyperparameter optimization results

Optimal hyperparameters identified during nested cross-validation for the finalized ExtraTrees classifier.

| **Outer fold** | **Optimal hyperparameters** | **Inner-fold ROC-AUC** |
| --- | --- | --- |
| Fold 1 | n_estimators=800; min_samples_leaf=2; max_features=sqrt; max_depth=25 | 0.8380 |
| Fold 2 | n_estimators=2000; min_samples_leaf=4; max_features=sqrt; max_depth=None | 0.8541 |
| Fold 3 | n_estimators=800; min_samples_leaf=2; max_features=sqrt; max_depth=25 | 0.8355 |
| Fold 4 | n_estimators=800; min_samples_leaf=2; max_features=sqrt; max_depth=25 | 0.8491 |
| Fold 5 | n_estimators=2000; min_samples_leaf=2; max_features=sqrt; | 0.8308 |

## Table S8. Summary of Y-randomization analysis

Summary statistics of label permutation analysis used to assess chance correlations.

| **Parameter** | **Value** |
| --- | --- |
| Number of label permutations | 499 |
| Trees per randomized model | 400 |
| Observed OOF ROC-AUC | 0.8584 |
| Null distribution mean ± s.d. | 0.4936 ± 0.0326 |
| Null distribution 95th percentile | 0.5463 |
| Empirical one-sided p-value | 0.002 |

**Note:** The null ROC-AUC distribution was centered near random expectation and substantially lower than the observed model performance.
